# *Nippostrongylus*-Induced Intestinal Hypercontractility Requires IL-4 Receptor Alpha-Responsiveness by T Cells in Mice

**DOI:** 10.1371/journal.pone.0052211

**Published:** 2012-12-20

**Authors:** Saskia Schmidt, J. Claire Hoving, William G. C. Horsnell, Helen Mearns, Antony J. Cutler, Tiroyaone Masego H. Bogale, Frank Brombacher

**Affiliations:** International Centre for Genetic Engineering and Biotechnology (ICGEB) and Division of Immunology, Institute of Infectious Disease and Molecular Medicine (IIDMM), Health Science Faculty, University of Cape Town, Cape Town, South Africa; CNRS, France

## Abstract

Gut-dwelling helminthes induce potent IL-4 and IL-13 dominated type 2 T helper cell (T_H_2) immune responses, with IL-13 production being essential for *Nippostrongylus brasiliensis* expulsion. This T_H_2 response results in intestinal inflammation associated with local infiltration by T cells and macrophages. The resulting increased IL-4/IL-13 intestinal milieu drives goblet cell hyperplasia, alternative macrophage activation and smooth muscle cell hypercontraction. In this study we investigated how IL-4-promoted T cells contributed to the parasite induced effects in the intestine. This was achieved using pan T cell-specific IL-4 receptor alpha-deficient mice (iLck^cre^IL-4Rα^−/lox^) and IL-4Rα-responsive control mice. Global IL-4Rα^−/−^ mice showed, as expected, impaired type 2 immunity to *N. brasiliensis*. Infected T cell-specific IL-4Rα-deficient mice showed comparable worm expulsion, goblet cell hyperplasia and IgE responses to control mice. However, impaired IL-4-promoted T_H_2 cells in T cell-specific IL-4Rα deficient mice led to strikingly reduced IL-4 production by mesenteric lymph node CD4^+^ T cells and reduced intestinal IL-4 and IL-13 levels, compared to control mice. This reduced IL-4/IL-13 response was associated with an impaired IL-4/IL-13-mediated smooth muscle cell hypercontractility, similar to that seen in global IL-4Rα^−/−^ mice. These results demonstrate that IL-4-promoted T cell responses are not required for the resolution of a primary *N. brasiliensis* infection. However, they do contribute significantly to an important physiological manifestation of helminth infection; namely intestinal smooth muscle cell-driven hypercontractility.

## Introduction

IL-4 and IL-13 share a common signalling pathway through the IL-4 receptor alpha (IL-4Rα) chain. A functional IL-4R (type I) requires assembly of IL-4Rα with a gamma c chain, while interaction of IL-4Rα with an IL-13Rα1 subunit leads to formation of a functional IL-13 receptor (type II). IL-4Rα–deficient mice lack responsiveness to IL-4 and IL-13. Expression of IL-4Rα reflects the pleiotropic nature of IL-4/IL-13 biology, as this receptor subunit is expressed upon a wide range of cells [1]. Mouse T and B lymphocytes lack the IL-13 receptor alpha 1 chain, hence T_H_2 differentiation and B cell isotype switching is dependent on IL-4 signalling via the type 1 IL-4Rα [2]. The transcription factors STAT-6 and GATA-3 are activated by IL-4Rα signalling to stabilize the T_H_2 program in polarized CD4^+^ T cells [1,3]. This leads to IgE and IgG1 antibody production [4,5] goblet cell hyperplasia [6] as well as secretion of cytokines IL-4, IL-13, IL-5, IL-10 and IL-9 [7].

In the gastrointestinal tract activated T_H_2 cells stimulate the production of IL-4 and IL-13 which enhances epithelial cell permeability [8] and leads to smooth muscle cell hypercontractility [9]. Together with goblet cell hyperplasia and increased mucus production [10], the intestinal hypercontractility causes a `weep and sweep response associated with the resolution of intestinal parasite infections [9,11]. Impaired *N. brasiliensis* expulsion occurs in mice deficient in STAT-6 [12,13], IL-13 [14], macrophages [15] or IL-4Rα [13,16] expression. Mechanistically, nematode expulsion requires goblet cell hyperplasia and has been associated with Relm-β expression by goblet cells [17,18]. Although intestinal hypercontractility has been associated with expulsion, this has not been conclusively demonstrated.

*N. brasiliensis* infection studies in experimental murine models are analogous to human hookworm infections [19]. These infections are characterised by IL-4Rα-driven responses which are essential for worm expulsion from the host intestine [13]. Recent helminth infection studies using global or smooth muscle cell-specific IL-4Rα deficient mice showed reduced intestinal contractility, which was concomitant with delayed worm expulsion [20,21]. Furthermore, *N. brasiliensis* infection resulted in impaired T_H_2 responses in global IL-4Rα and smooth muscle cell-specific IL-4Rα deficient BALB/c mice and accompanied by delayed goblet cell hyperplasia in these mice [20]. Together, these results indicate that a coordinated T_H_2 response may contribute to smooth muscle cell contraction. In contrast, macrophage/neutrophil-specific IL-4Rα deficient mice, which have impaired IL-4Rα-activated alternative macrophages [22]–[27], developed protective immunity against *N. brasiliensis* infection accompanied by goblet cell hyperplasia.

Our previous studies have shown that the expression of IL-4Rα specifically on CD4^+^ T cells and macrophage/neutrophils is not required for *N. brasiliensis* expulsion [24,28]. In this study, we used recently established pan (CD4^+^, CD8^+^, NK T and γδ) T cell-specific IL-4Rα (iLck^cre^IL-4Rα^−/lox^) deficient mice [29] and demonstrated that IL-4Rα expression by T cells is also not required for worm expulsion. Furthermore, we showed evidence that IL-4Rα responsiveness by T cells is needed for IL-4/IL-13-mediated intestinal hypercontractility.

## Methods

### Ethics Statement

All experiments were approved by the University of Cape Town Animal Ethics Committee (approval number 008/019) and all efforts were made to minimize suffering.

### Mice

Eight- to 12-week-old mice were obtained from the University of Cape Town specific-pathogen-free animal facility and kept in individually ventilated cages. T cell- (iLck^cre^IL-4Rα^−/lox^) IL-4Rα deficient mice were generated as previously described [29] and hemizygous IL-4Rα^−/lox^ mice (littermate control mice) and homozygous IL-4Rα^−/−^ mice (IL-4Rα KO mice) were used as controls. iLck^cre^IL-4Rα^−/lox^ mice are described as *C.Cg-Il4ra^tm1Fbb^/Il4ra^tm2Fbb^Tg(Lck-cre)*, and IL-4Rα^−/−^ are *Il4ra^tm1Fbb^/Il4ra^tm1Fbb^*. All mice used were on a BALB/c background. In addition, BALB/c mice were compared to hemizygous IL-4Rα^−/lox^ mice and improved iLck^cre^IL-4Rα^−/lox^ compared with Lck^cre^IL-4Rα^−/lox^ mice [30].

### *N. brasiliensis* Infection

Mice were inoculated subcutaneously with 750 *N. brasiliensis* L3 larvae. An analysis of parasite eggs in faeces was carried out using the modified McMaster technique [31]. Adult worm burdens were determined as previously described [16]. Briefly, intestines were removed from infected mice, and each lumen was exposed by dissection. The intestines were then incubated at 37°C for 4 h in 0.65% NaCl. Intestinal tissue was then removed, and the adult worms in the remaining saline solution were counted.

### Histology

Tissue samples were fixed in a neutral buffered formalin solution. Following embedding in paraffin, samples were cut into 5-µm sections. Sections were stained with periodic acid-Schiff reagent (PAS) for quantification of intestinal goblet cell hyperplasia, which was carried out as previously described [20,32]. Briefly, intestinal goblet cell hyperplasia in individual mice was determined by counting the number of positive goblet cells per five villi from the small intestine. Smooth muscle layer thickness was measured in haematoxylin and eosin stained sections from individual mice. Essentially, Nikon NIS elements software was used to measure the thickness and the mean of ±40 measurements per mouse was plotted for days 3, 7 and 10 PI.

### Enzyme-linked Immunosorbent Assay (ELISA) Analysis

CD4^+^ T-cells isolated by negative selection using Biomag beads (Qiagen) with a purity of >90% [as previously described 20] were restimulated for 48 h with 20 µg/ml anti-CD3 antibody 145-2C11. Supernatants were then collected and stored at −80°C until they were analyzed. Cytokine levels in supernatants and total IgE antibody levels in serum of individual infected animals were determined as previously described [33]. Briefly, flat bottom 96-well plates were coated overnight with the appropriate capturing antibody diluted in PBS (IgE clone 84.1C; IL-4 clone 11B11; IL-13 clone 38213.11, IFN-g clone An18KL6, IL-17 clone 50101). The plates were then washed and incubated in PBS containing 2% milk for 1 h at 37°C. Following this, the plates were washed, and samples and standards were loaded overnight at 4°C. Appropriate biotinylated secondary antibodies were then added following further washing and incubated overnight at 4°C (IgE clone 23G3; IL-4 clone BVD6-24G2; IL-13 clone TRFK4; IFN-g clone XMG1.2, IL-17). The plates were then washed, and antibody and cytokine levels were determined using streptavidin-coupled horseradish peroxidase. The plates were developed with a 3,3,5,5-tetramethylbenzidine microwell peroxidase substrate system, and the reaction was stopped with 1 M H_3_PO_3_. The absorbance at 450 nm was determined with a Versamax microplate spectrophotometer (Molecular Devices, Germany). Total IgE >0.002 ug/ml and IL-4, IL-13 or IFN-g >0.412 ng/ml were detected.

For intestinal cytokine detection the jejunum was removed from naive and infected mice and homogenized in lysis buffer containing protease inhibitors (Sigma). The homogenates were centrifuged at 14000 rpm for 20 min and the protein concentration in the supernatant was determined using the BCA assay (Pierce, Rockford IL). Protein concentration for all samples were equalised to 3 mg/ml and the levels of the cytokines IL-4 and IL-13 were determined using ELISA (see above).

### Measurement of Intestinal Contractility

Whole tissue sections, 1 cm long were dissected from the jejunum region of the small intestine and suspended in a four chamber automatic organ bath system in oxygenated Krebs buffer at a resting tension of 0.5 g as previously described [34]. Data acquisition and analysis was conducted by the ADInstruments Powerlab® and the LabChart® analysis software. In brief all tissue was weighed, stimulated with 50 mM potassium chloride (KCl) prior to acetylcholine (−9 to −3 LOG[M]) stimulation, washed and equilibrated for 10 min between each dose, and contractile force expressed in mN/mg of tissue.

### Statistics

Values are expressed below as means ± standard deviations or means ± standard errors of the means, and significant differences were determined using the Mann-Whitney U test, an unpaired two-tailed Student t test or a One-Way ANOVA (GraphPad Prism4).

## Results

### Expulsion of *N. brasiliensis* is not Dependent on IL-4Rα-responsive T Cells

**Figure 1:**
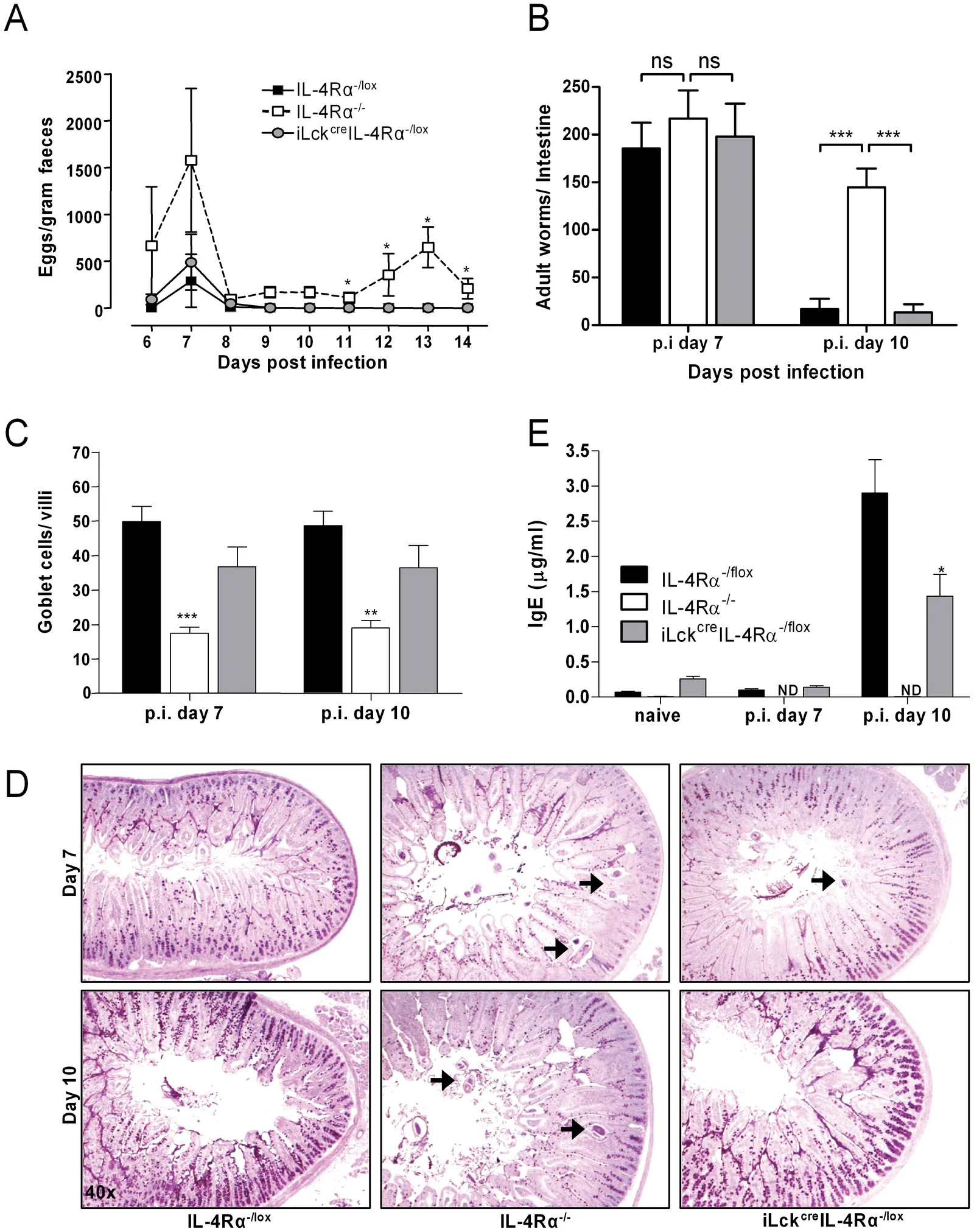
IL-4 responsive T cells are not needed for expulsion of *N. brasiliensis.* iLck^cre^IL-4Rα^−/lox^ and control mice were infected with 750 *N. brasiliensis* L3 larvae. Faeces were collected from day 6 to 14 post infection (PI) and egg production was calculated using the modified McMaster technique (A). At days 7 and 10 PI the worm burden in the small intestine was assessed (pooled from 3 experiments) (B). Intestinal goblet cell hyperplasia was assessed by determining the total number of PAS-positive goblet cells per 5 villi in histological sections of the small intestine at day 7 and 10 PI (C). Mucus and PAS staining at days 7 and 10 PI. Representative photomicrographs are shown from individual mice and *N. brasiliensis* is indicated with a black arrow (D). Total IgE production in the serum was measured by ELISA at day 7 and 10 PI (E). The graphs show mean values ± SEM and represent the results of three independent experiments, except B and E where 2–3 independent experiments were combined with n = 4 or 5 mice per group. ND, not detected. One-Way-ANOVA, *P<.05, **P<.01, ***P<.001 for all experiments.

To investigate the role of IL-4Rα-responsive T cells in the control of *N. brasiliensis* infection, IL-4Rα^−/−^, pan T cell-specific IL-4Rα deficient (iLck^cre^IL-4Rα^−/lox^) and heterozygous IL-4Rα^−/lox^ littermate control mice were infected by subcutaneous injection of 750 L3 *N. brasiliensis* larvae. Parasite egg production was determined on days 6–14 PI (Figure 1 A) and intestinal adult worm burdens determined on days 7 and 10 PI (Figure 1B and Figure S1). Heterozygous IL-4Rα^−/lox^ control and iLck^cre^IL-4Rα^−/lox^ mice showed similar egg production throughout the infection with egg counts peaking at day 7 and clearing by day 9 post infection (PI). Intestinal worm burdens in both mice strains were similar at day 7 PI and absent by day 10 PI. As previously demonstrated, IL-4Rα^−/−^ mice did not clear infection efficiently showing a maintained egg production at day 11 PI and the presence of adult worms detected at day 10 PI [20,24]. As seen in previously described CD4^+^ T cell-specific IL-4Rα deficient mice (Lck^cre^IL-4Rα^−/lox^) [28], pan T cell-specific IL-4Rα deficient mice efficiently clear the worms similar to IL-4Rα-responsive control mice.

T cell subpopulations other than CD4^+^ T cells are also known to play a key role in *N. brasiliensis* clearance, such as γδ T cells which initiate rapid expulsion of adult worms from the intestine and limit egg production [35]. To determine if IL-4Rα surface expression on CD8^+^, γδ and NK T cells plays a role in *N. brasiliensis* expulsion we compared pan T cell-specific IL-4Rα deficient mice (iLck^cre^IL-4Rα^−/lox^) described in this paper with the previously described CD4^+^ T cell-specific IL-4Rα deficient (Lck^cre^IL-4Rα^−/lox^) mice which have partial or normal IL-4Rα surface expression on CD8^+^, γδ and NK T cells (Table S1). Both strains showed comparable worm expulsion, egg numbers and IL-13 production (Figure S2 A–E) therefore we concluded that IL-4Rα-responsive T cells are not crucial for *N. brasiliensis* expulsion.

Furthermore, to determine the influence of loxP insertion on IL-4 receptor function we compared *N. brasiliensis* infected WT BALB/c mice with heterozygous IL-4Rα^−/lox^ control mice and found no difference (Figure S3 A–E). These results suggest that neither loss of one IL-4Rα allele nor silent mutation due to lox insertion has a significant effect on acetylcholine-mediated contraction.

### Normal Intestinal Goblet Cell Hyperplasia in Infected T Cell-specific IL-4Rα Deficient Mice

**Figure 2:**
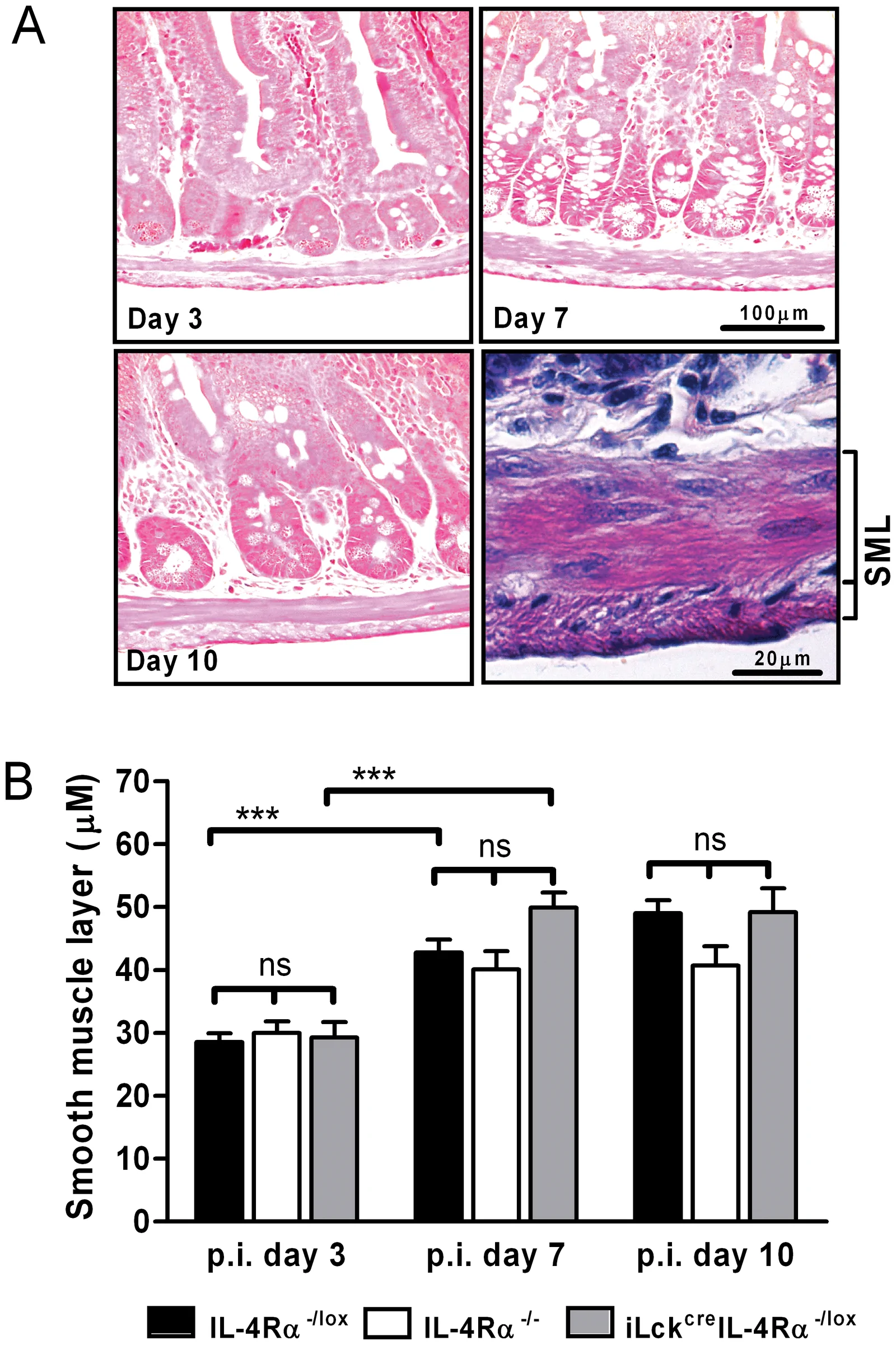
*N. brasiliensis* induced smooth muscle cell hypertrophy/hyperplasia is unaffected in iLck^cre^IL-4Rα^−/lox^ mice. Haematoxylin and eosin stained sections were used to determine the smooth muscle cell layer thickness from Day 3, 7 and 10 *N. brasiliensis-*infected iLck^cre^IL-4Rα^−/lox^ and control mice. Representative photomicrographs are shown from control mice at days 3, 7 and 10 at 40× magnification. Also shown is a photomicrograph at 200× showing the longitudinal and circular smooth muscle layers included in the measurement (A). Measurements are shown in a bar graph (B) with mean values+SEM and represent 2 independent experiments with n = 4 or 5 mice per group. Ns = not significant. One-Way-ANOVA, ***P<.001.

A key host response induced and associated with expulsion of adult *N. brasiliensis* from the intestine is increased IL-4Rα-dependent goblet cell hyperplasia and mucus production (16). Quantification of PAS-stained mucus-containing goblet cells in the small intestine resulted in similar number per villi between control and iLck^cre^IL-4Rα^−/lox^ mice (Figure 1C and D) with significantly lower intestinal mucus production in global IL-4Rα^−/−^ mice, (as previously shown) (20,24). Whereas total IgE antibody concentration was below detection limit in the sera of global IL-4Rα^−/−^ mice, IgE antibodies were present in naive iLck^cre^IL-4Rα^−/lox^ mice and increased during infection, though to a lesser extent than infected control mice (Figure 1E). Together, this indicates that sufficient IL-4 is present for IL-4Rα-dependent type 2 B-cell responses. As *N. brasiliensis* is known to cause intestinal smooth muscle hyperplasia/hypertrophy we measured the thickness of this layer in the intestine of all mouse groups. Indeed we detected a significant thickening of this muscle layer when comparing day 3 (before the worms have reached the intestine) with day 7 and 10 post infection (Figure 2A and B). However, there was no significant difference between all mouse groups suggesting that the thickening is independent of IL-4Rα.

### IL-4 and IL-13 Production in the Jejunum is Abrogated in Infected T Cell-specific IL-4Rα Deficient Mice

**Figure 3:**
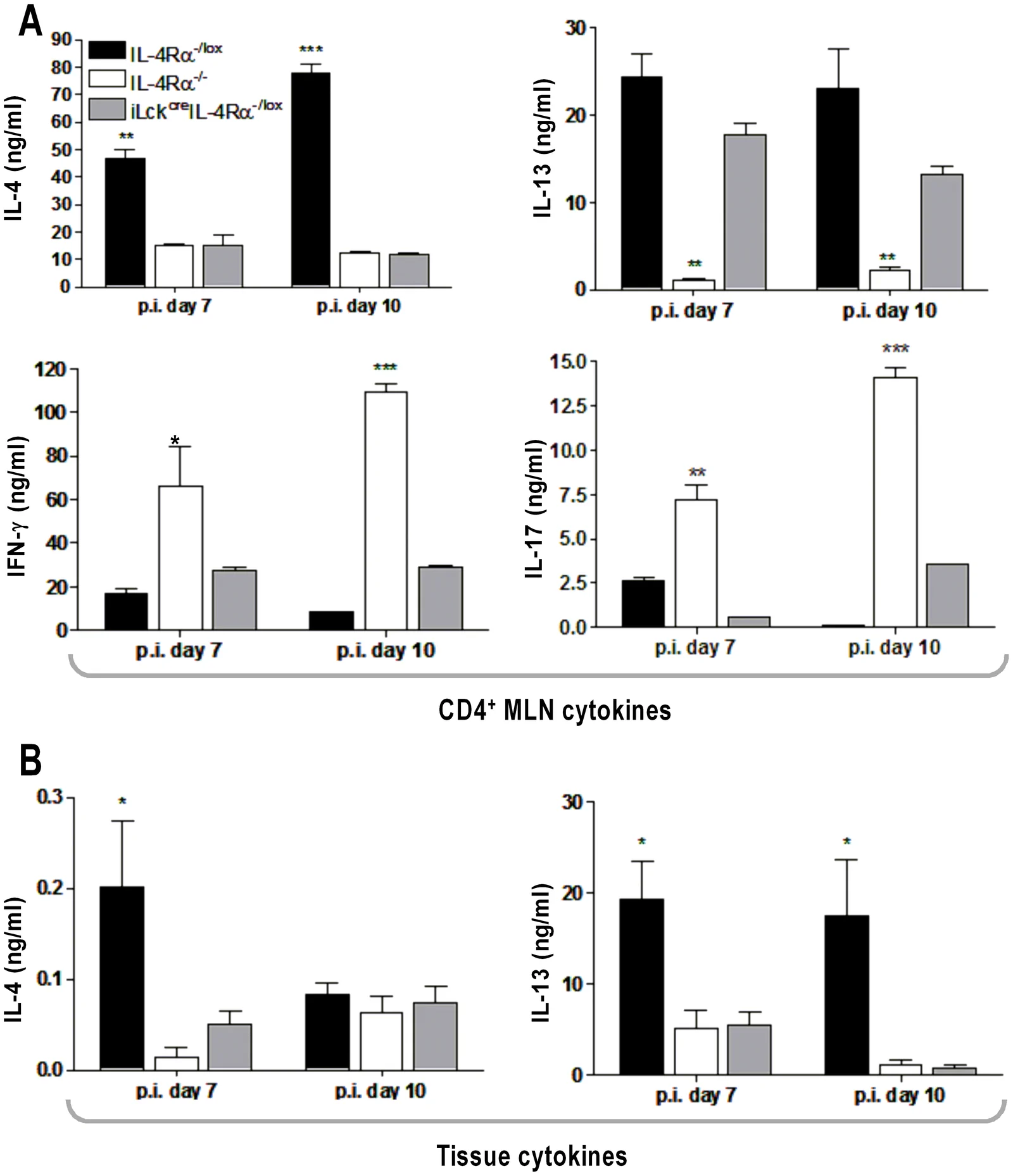
Reduced IL-4 response in *N. brasiliensis-*infected iLck^cre^IL-4Rα^−/lox^ and IL-4Rα^−/−^ mice. Mice were infected with 750 *N. brasiliensis* L3 larvae and at days 7 and 10 PI CD4^+^ cells from pooled mesenteric lymph nodes were isolated by negative selection (purity>90%) then restimulated with anti-CD3 for 48 hours and IL-4, IL-13, INF-γ, IL-17 cytokine concentration of the supernatant determined by ELISA (A). Further, IL-4 and IL-13 concentrations were determined in homogenates of the jejunum (B). The graphs show mean values+SEM and are representative of the results of three independent experiments with IL-17 only determined in one experiment for CD4^+^ T cells and IL-13 in two independent experiments for homogenates, with n = 4 or 5 per group. One-Way-ANOVA, *P<.05, **P<.01, ***P<.001.

In order to determine T helper cytokine responses, mesenteric lymph node CD4^+^ T cells were isolated at days 7 and 10 PI, then restimulated with anti-CD3. As expected, IL-4Rα-responsive CD4^+^ T cells from IL-4Rα^−/lox^ control mice secreted high levels of the T_H_2 cytokines, IL-4 and IL-13, reduced T_H_1 associated IFN-γ and T_H_17 associated IL-17 when compared to the IL-4Rα-unresponsive CD4^+^ T cells from IL-4Rα^−/−^ mice (20,24) (Figure 3A). Interestingly, CD4^+^ T cells derived from iLck^cre^IL-4Rα^−/lox^ mice showed a similar reduction of IL-4 as CD4^+^ T cells derived from IL-4Rα^−/−^ mice). However, IL-13 and IL-17 secretion by iLck^cre^IL-4Rα^−/lox^ mice was not significantly different to control mice (Figure 3A). Together, these data suggest that IL-4 but not IL-13 responses require IL-4-promoted T_H_2 cells during *N. brasiliensis* infection in CD4^+^ T cells from mesenteric lymph nodes.

To determine levels of IL-4 and IL-13 in the jejunum, soluble homogenates of tissue were analysed by ELISA. As expected, *N. brasiliensis* infection induced the T_H_2 cytokines IL-4 and IL-13 in the jejunum of IL-4Rα^−/lox^ control mice (Figure 3B). In contrast, T cell-specific IL-4Rα deficient mice showed impaired IL-4 and IL-13 cytokine response of equivalent magnitude to IL-4Rα^−/−^ mice. These results are supported by our previous study where mediastinal lymph node CD4^+^ T cells from mice lacking IL-4Rα expression specifically on CD4^+^ T cells (Lck^cre^IL-4Rα^−/lox^) maintained their ability to produce IL-13 in contrast to the CD4^+^ T cells isolated from digested lung [28]. Together these results demonstrate impaired IL-4 production by mesenteric CD4^+^ T cells and impaired IL-4 and IL-13 levels in the jejunum of *N. brasiliensis*-infected T cell-specific IL-4Rα deficient mice.

### *N. brasiliensis* Induced Hypercontractility is Impaired in Infected T Cell- specific IL-4Rα Deficient Mice

**Figure 4:**
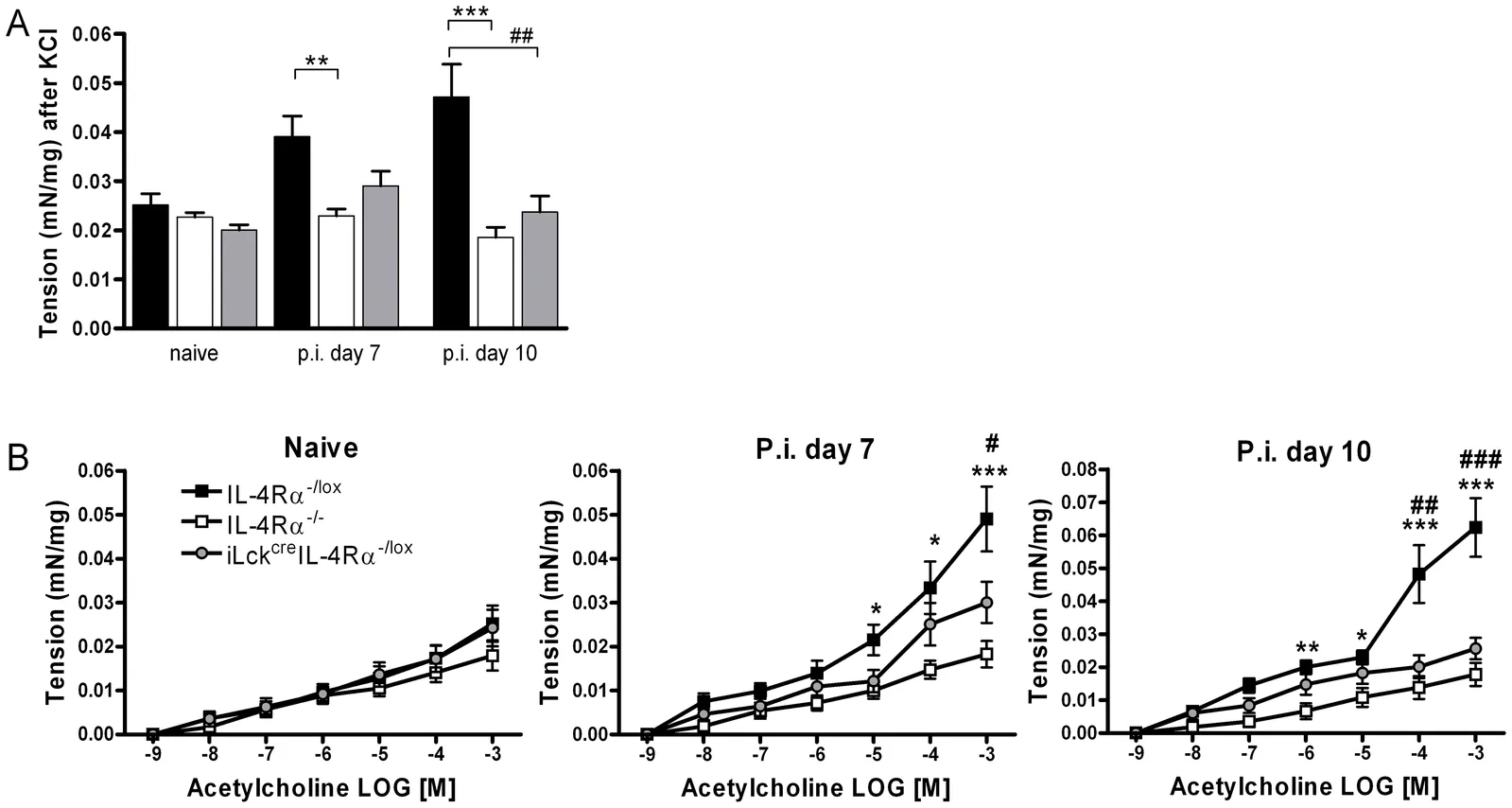
*N. brasiliensis*-induced and IL-4Rα-mediated intestinal hypercontractility is impaired in iLck^cre^IL-4Rα^−/lox^ mice. Jejunum pieces (1 cm) of non-infected and *N. brasiliensis* infected (PI day 7 and 10) mice were stimulated with KCl and contractility was measured (A). Comparison of the different mouse strains in response to acetylcholine is also shown for naïve, day 7 or day 10 infected IL-4Rα^−/lox^, IL-4Rα^−/−^ and iLck^cre^IL-4Rα^−/lox^ mice (B). Contractility is shown as a mean value ± SEM for individual dose points. Graphs show three independent experiments with n = 12 in total. One-Way-ANOVA, *,# *P<.05*; **,## *P<.01*; ***,### *P<.001*. *indicates statistical significant differences between IL-4Rα^−/lox^ and IL-4Rα^−/−^, # shows differences between IL-4Rα^−/lox^ and iLck^cre^IL-4Rα^−/lox^ mice.

Recently, we described that nematode infection induced an IL-4/IL-13-driven intestinal smooth muscle hypercontractility, which was absent in global IL-4Rα^−/−^ mice and reduced in smooth muscle cell-specific IL-4Rα^−/−^ mice [21]. To determine if IL-4 responsive T cell responses contributed to intestinal smooth muscle cell hypercontractility, *ex vivo* contractile ability of jejunum from infected iLck^cre^IL-4Rα^−/lox^ mice was compared to control IL-4Rα^−/lox^ and global IL-4Rα^−/−^ mice after 7 or 10 days PI. Jejunum weight was equivalent between all strains under naive conditions and at 7 days PI, while at day 10 PI the tissue weight was increased in the global IL-4Rα^−/−^ but not in iLck^cre^IL-4Rα^−/lox^ mice compared to controls (data not shown). Jejunum contractile responses to stimulation with potassium chloride and acetylcholine in naïve mice were similar in all groups (Figure 4A). Following infection (day 7 and 10) contractile responses significantly increased in control mice but not global IL-4Rα^−/−^ mice. Importantly, in iLck^cre^IL-4Rα^−/lox^ mice the hypercontractile response was also significantly reduced at day 10 PI. The described enhanced potassium chloride induced intestinal contractility in control mice after *N. brasiliensis* infection has been previously described in *Schistosoma mansoni* infection and is suggested to be caused by non-ligand specific hypercontractions [36,37]. Our findings indicate that optimal KCL induced intestinal responses require IL-4Rα expression.

As previously shown [21], infection with *N. brasiliensis* enhanced tension to acetylcholine significantly in IL-4Rα-responsive control mice when compared to non-infected control mice (Figure 4B). As expected, jejunum from infected global IL-4Rα^−/−^ mice did not hypercontract in response to acetylcholine. Comparison of the IL-4Rα-responsive control and global IL-4Rα^−/−^ mice, with iLck^cre^IL-4Rα^−/lox^ mice showed no tension differences under naive conditions. However, infection with *N. brasiliensis* showed increased tension at day 7 and 10 in control IL-4Rα^−/lox^ mice compared to global IL-4Rα^−/−^ and iLck^cre^IL-4Rα^−/lox^ mice. Together, these results show that IL-4Rα responsive T cells are needed for optimal *N. brasiliensis*-induced smooth muscle cell hypercontractility.

## Discussion

Morphological and physiological changes in the gastrointestinal system during nematode infections may be important contributors to host defence and pathology. These responses have previously been demonstrated to be controlled by the T_H_2 immunity associated with infection. Non-haematopoietic contributions by IL-4Rα responsive smooth muscle cells have been previously demonstrated [12]. It is however important to understand the molecular requirements of haematopoietic cell populations to contribute to this striking physiological response. Using a mouse model with impaired IL-4Rα expression on specific T cell populations, we demonstrated roles for IL-4 responsive T cells in intestinal hypercontractile responses to *N. brasiliensis.* In this study the impact of IL-4Rα-responsive T cells on smooth muscle cell hypercontraction and their contribution to clearance of *N. brasiliensis* infection was defined. We showed that IL-4Rα-responsive T cells are required for optimal *N. brasiliensis*-induced intestinal hypercontractility, but not for worm expulsion.

Wild type mice resist infection with *N. brasiliensis* and develop polarized T_H_2 responses with high IL-4/IL-13 and low IFN-γ production [38–40]. Well-established T_H_2 induced effector mechanisms following *N. brasiliensis* infection are eosinophilia [4,41] mucosal mastocytosis [6], pathogen specific antibodies including IgE and IgG1 [4,5] goblet cell hyperplasia and promotion of T_H_2 cytokine responses. IL-4 has been implicated in driving the polarized T_H_2 response against *N. brasiliensis,* demonstrated by diminished type 2 responses in IL-4^−/−^, IL-4Rα^−/−^ and STAT-6^−/−^ mice [16,24,28,37,42,43]. Although it is known that both IL-4Rα [13] and CD4^+^ T cells [20] are involved in worm clearance, more recent studies from us and others have shown that IL-4 responsive CD4^+^ T cells [28] or signalling through the STAT-6 pathway in these cells is not needed for worm expulsion. Indeed, IL-4 receptor expression by non-bone marrow-derived cells is required to expel *N. brasiliensis* [44]. However, type 2 immunity is controlled by IL-4/IL-13 expression in haematopoetic non-eosinophil cells of the innate immune system [45]. This is consistent with the findings in our present study, as infected mice deficient in IL-4Rα expression on all T cell subpopulations showed impaired T_H_2 responses but still presentedIL-13 production in the mesenteric lymph nodes, able to respond with goblet cell hyperplasia and controlling infection. In contrast, global IL-4Rα mice could not respond to IL-4 or IL-13, hence were impaired in effective worm expulsion. IL-4 is also known to suppress T_H_17 development in a STAT-6 dependent manner [46] with IL-4Rα^−/−^ mice producing increased levels of IL-17 in an allergic asthma model [47]. We showed that IL-17 production is increased in IL-4Rα^−/−^ mice in response to *N. brasiliensis* but remains comparable with control mice in iLck^cre^IL-4Rα^−/lox^ mice. This suggests that the suppression of IL-17 is independent of IL-4Rα signalling on T cells.

Recent research showed that infection with different nematodes induces an increased smooth muscle cell driven intestinal contractility in wild-type mice [9,15,21,33]. This is believed to be instrumental for the weep and sweep process to diminish the worm from the gut lumen. It has been shown that IL-4 and IL-13 promote acetylcholine-induced intestinal hypercontractility and that IL-4 can directly enhance smooth muscle cell contractility without influencing the enteric nervous system [9]. Moreover, responses to acetylcholine were attenuated in STAT6^−/−^ mice, which suggest at least a partial dependence of smooth muscle cell hypercontractility on the IL-4/IL-13/STAT-6 pathway [9]. This was recently substantiated by us as the jejunum of *N. brasiliensis*-infected smooth muscle cell-specific IL-4Rα deficient mice, and more drastically *N. brasiliensis*-infected global IL-4Rα^−/−^ mice showed abrogated contractility in response to acetylcholine stimulation [21]. Little is known about the possible role of other cell types in worm-induced intestinal smooth muscle cell hypercontraction. However, it has been shown that macrophages play a key role in negatively regulating *Trichinella spiralis* induced hypercontractility, which is in-part mediated through macrophage M-CSF production [48,49]. In this study, we showed that IL-4Rα-responsive T cells are needed for efficient intestinal smooth muscle cell contraction. Absence of IL-4-responsive T cells resulted in impaired IL-4 production from CD4^+^ T cells in the mesenteric lymph node and strikingly reduced IL-4 and IL-13 production in the intestine, which explains impaired IL-4/IL-13-mediated smooth muscle cell hypercontractility. Interestingly, mice were still able to expel the worm despite abrogated intestinal hypercontractility.

In conclusion, this study highlights the contributing role of IL-4-promoted T_H_2 cells with their major importance not in worm expulsion but in controlling IL-4/IL-13-induced intestinal hypercontractility. Although this is a major host physiological response to helminthes, it seems that smooth muscle hypercontractility induced by acetylcholine is not needed for efficient worm expulsion during primary *N. brasiliensis* infection.

## Supporting Information

Figure S1 IL-4 responsive T cells are not needed for expulsion of *N. brasiliensis.* Duplicated worm burdens from figure 1B represented as individual counts at days 7 and 10 PI. As above, the data represents three independent experiments combined, with n = 4 or 5 per group, ns = not significant. One-Way-ANOVA, ***P<.001.
(TIF)

Figure S2 *N. brasiliensis* infection is comparable between iLck^cre^IL-4Rα^−/lox^ and Lck^cre^IL-4Rα^−/lox^ mice. iLck^cre^IL-4Rα^−/lox^, Lck^cre^IL-4Rα^−/lox^ and control mice were infected with 750 *N. brasiliensis* L3 larvae. Faeces were collected from day 5 to 10 post infection (PI) and egg production was calculated using the modified McMaster technique (A). At days 7 and 10 PI the worm burden in the small intestine was assessed (B). Intestinal goblet cell hyperplasia was assessed by determining the total number of PAS-positive goblet cells per 5 villi in histological sections of the small intestine at day 7 and 10 PI (C). Total IgE production in the serum was measured by ELISA at day 7 and 10 PI (D). The data are representative of the results of two independent experiments with mean values+SEM and n = 4 or 5 mice per group. ND, not detected, ns = not significant. One-Way-ANOVA, *P<.05, **P<.01.
(TIF)

Figure S3 *N. brasiliensis* infection is comparable between BALB/c and IL-4Rα^−/lox^ mice. Five mice per group were infected with 750 *N. brasiliensis* L3 larvae. Faeces were collected from day 5 to 10 post infection (PI) and egg production was calculated using the modified McMaster technique (A). At days 7 and 10 PI the worm burden in the small intestine was assessed (B). Intestinal goblet cell hyperplasia was assessed by determining the total number of PAS-positive goblet cells per 5 villi in histological sections of the small intestine at day 7 and 10 PI (C). Total IgE production in the serum was measured by ELISA at day 7 and 10 PI (D). Comparison of the response of infected BALB/c and IL-4Rα^−/lox^ mice to acetylcholine is also shown for day 7 p.i. The data represents one (A-D) and two (E) independent experiment with n = 5 per group and mean values + SEM. ND, not detected. Unpaired two-tailed Student t test, ns = not significant.
(TIF)

Table S1 Summary of IL-4Rα surface expression on T cell subpopulations. Table S1 summarizes the surface expression of IL-4Rα on T cell subpopulations determined by FACS as previously described [29,30]. Subpopulations include CD4^+^, CD8^+^, γδ T cells and NK T cells.
(DOC)
