# Supplementary figures and images for: Nippostrongylus-Induced Intestinal Hypercontractility Requires IL-4 Receptor Alpha-Responsiveness by T Cells in Mice
Source: PLoS One. 2012 Dec 20;7(12):e52211. doi: 10.1371/journal.pone.0052211 (PMC3527412; doi:10.1371/journal.pone.0052211)

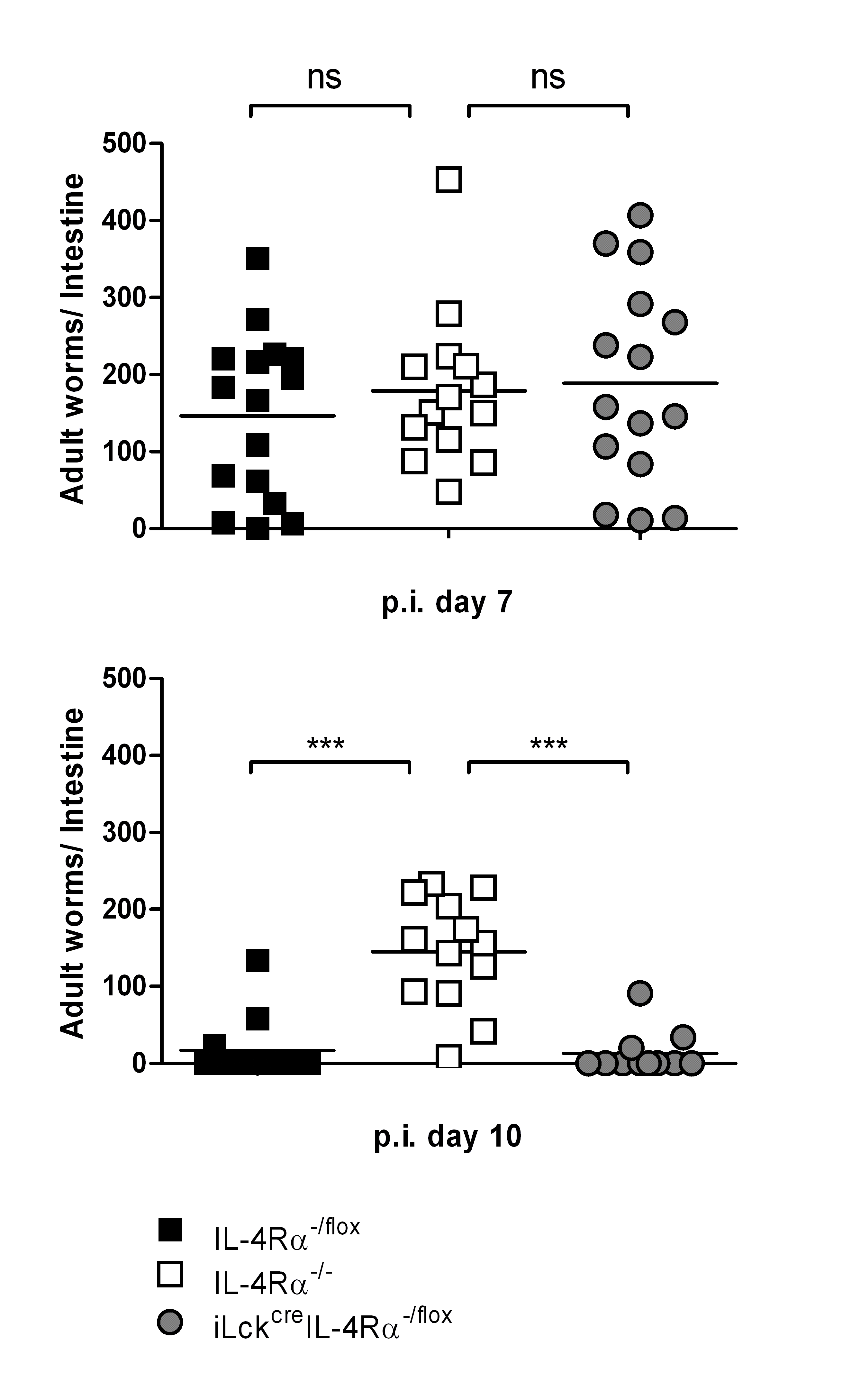

Supplement: Figure S1 — IL-4 responsive T cells are not needed for expulsion of N. brasiliensis. Duplicated worm burdens from figure 1B represented as individual counts at days 7 and 10 PI. As above, the data represents three independent experiments combined, with n = 4 or 5 per group, ns = not significant. One-Way-ANOVA, ***P<.001. (TIF) [file pone.0052211.s001.tif]

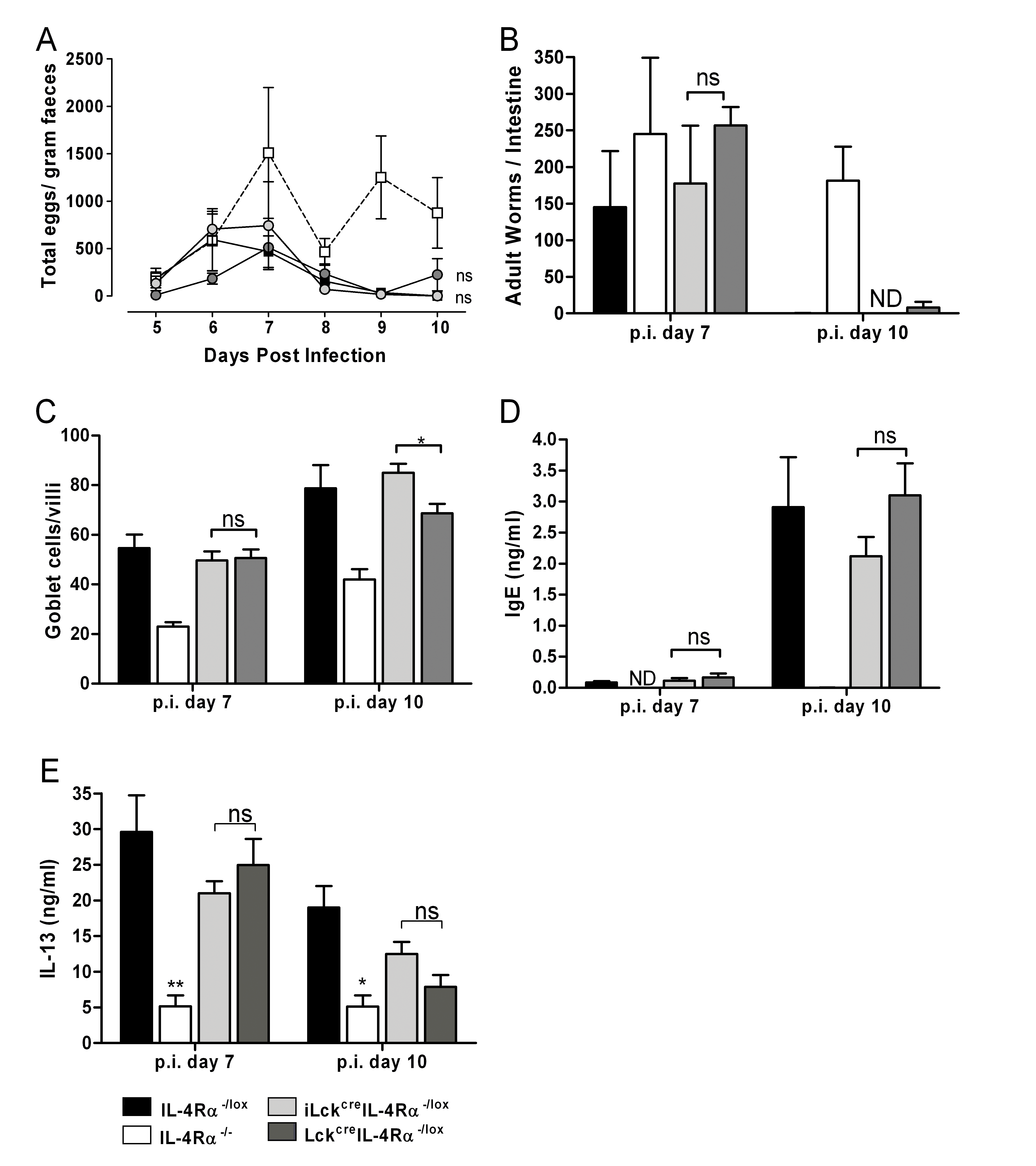

Supplement: Figure S2 — N. brasiliensis infection is comparable between iLckcreIL-4Rα−/lox and LckcreIL-4Rα−/lox mice. iLckcreIL-4Rα−/lox, LckcreIL-4Rα−/lox and control mice were infected with 750 N. brasiliensis L3 larvae. Faeces were collected from day 5 to 10 post infection (PI) and egg production was calculated using the modified McMaster technique (A). At days 7 and 10 PI the worm burden in the small intestine was assessed (B). Intestinal goblet cell hyperplasia was assessed by determining the total number of PAS-positive goblet cells per 5 villi in histological sections of the small intestine at day 7 and 10 PI (C). Total IgE production in the serum was measured by ELISA at day 7 and 10 PI (D). The data are representative of the results of two independent experiments with mean values+SEM and n = 4 or 5 mice per group. ND, not detected, ns = not significant. One-Way-ANOVA, *P<.05, **P<.01. (TIF) [file pone.0052211.s002.tif]

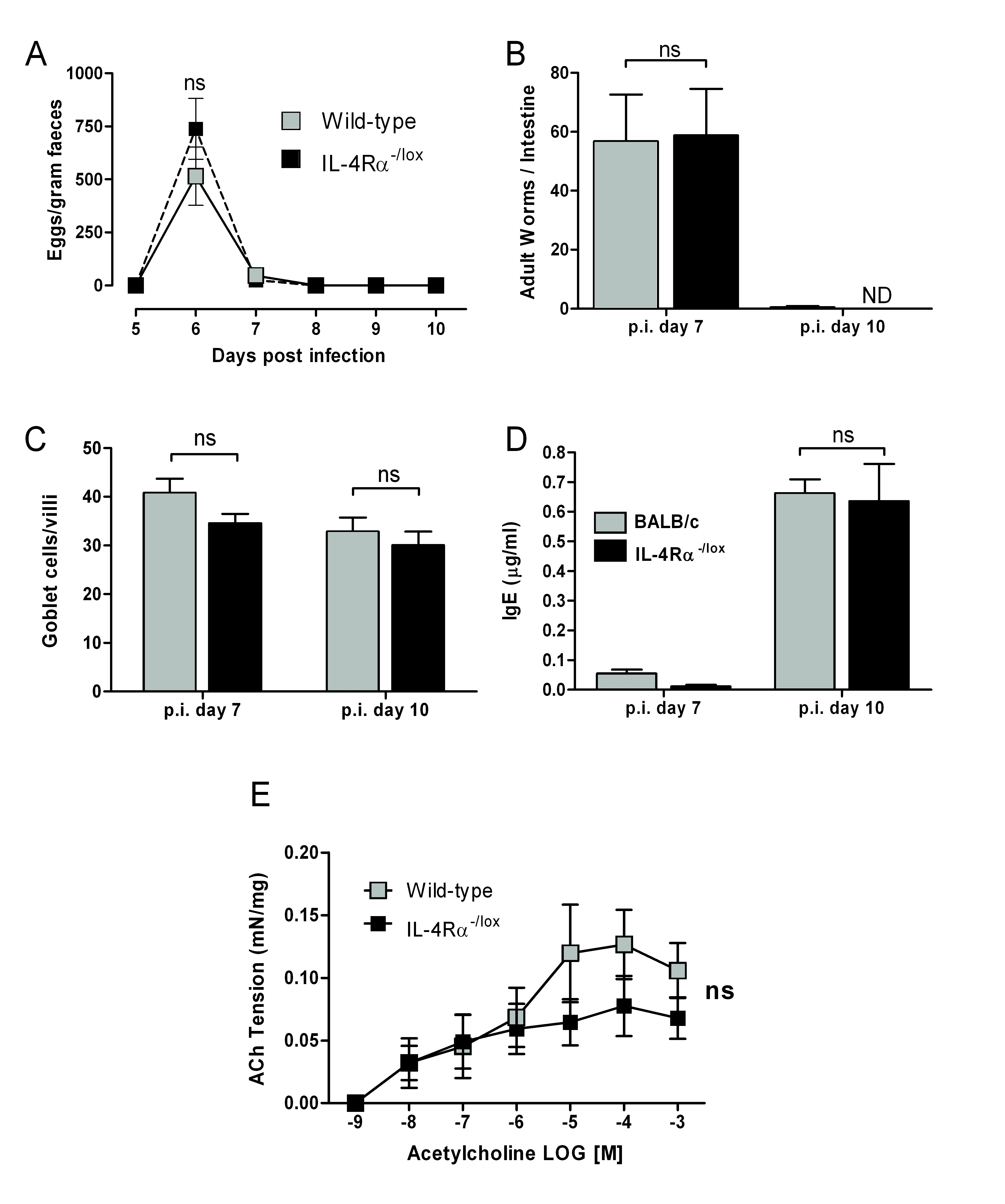

Supplement: Figure S3 — N. brasiliensis infection is comparable between BALB/c and IL-4Rα−/lox mice. Five mice per group were infected with 750 N. brasiliensis L3 larvae. Faeces were collected from day 5 to 10 post infection (PI) and egg production was calculated using the modified McMaster technique (A). At days 7 and 10 PI the worm burden in the small intestine was assessed (B). Intestinal goblet cell hyperplasia was assessed by determining the total number of PAS-positive goblet cells per 5 villi in histological sections of the small intestine at day 7 and 10 PI (C). Total IgE production in the serum was measured by ELISA at day 7 and 10 PI (D). Comparison of the response of infected BALB/c and IL-4Rα−/lox mice to acetylcholine is also shown for day 7 p.i. The data represents one (A-D) and two (E) independent experiment with n = 5 per group and mean values + SEM. ND, not detected. Unpaired two-tailed Student t test, ns = not significant. (TIF) [file pone.0052211.s003.tif]
