# Supplementary material for: Nippostrongylus-Induced Intestinal Hypercontractility Requires IL-4 Receptor Alpha-Responsiveness by T Cells in Mice
Source: PLoS One. 2012 Dec 20;7(12):e52211. doi: 10.1371/journal.pone.0052211 (PMC3527412; doi:10.1371/journal.pone.0052211)
Supplement: Table S1 — Summary of IL-4Rα surface expression on T cell subpopulations. Table S1 summarizes the surface expression of IL-4Rα on T cell subpopulations determined by FACS as previously described [29], [30]. Subpopulations include CD4+, CD8+, γδ T cells and NK T cells. (DOC) [file pone.0052211.s004.doc]

**Table S1: Summary of IL-4Rα** surface expression on T cell subpopulations

|  | **CD4+** | **CD8+** | **γδ T cell** | **NK T cell** |
| --- | --- | --- | --- | --- |
| **WT** | Expressed | Expressed | Expressed | Expressed |
| **IL-4Rα**^-/-^ | Deleted | Deleted | Deleted | Deleted |
| **iLck^cre^IL-4Rα^-/flox (29)^** | Deleted | Deleted | Deleted | Deleted |
| **Lck^cre^IL-4Rα^-/flox (30)^** | Deleted | Partial | Expressed | Partial |
